# German translation and psychometric testing of the Postconcussion Symptom Inventory for adolescents in self-report (PCSI-SR13) and parent-report (PCSI-P)

**DOI:** 10.1371/journal.pone.0307421

**Published:** 2025-08-08

**Authors:** Dagmar A. Timmermann, Marina Zeldovich, Ugne Krenz, Ivana Holloway, Katrin Cunitz, Inga K. Koerte, Anna Buchheim, Christel Salewski, Gerard A. Gioia, Nicole von Steinbuechel

**Affiliations:** 1 Division Medical Psychology and Medical Sociology, Department of Psychosomatic Medicine and Psychotherapy, University Medical Center Goettingen, Goettingen, Germany; 2 Department of Psychology, University of Innsbruck, Innsbruck, Austria; 3 Faculty of Psychotherapy Science, Sigmund Freud University Vienna, Vienna, Austria; 4 Institute of Medical Psychology and Medical Sociology, University Medical Center Goettingen, Goettingen, Germany; 5 Department of Clinical and Population Sciences, School of Medicine, Leeds Institute for Data Analytics, Leeds, United Kingdom; 6 cBRAIN, Department of Child and Adolescent Psychiatry, Psychosomatics and Psychotherapy, Ludwig-Maximilians-Universität, Munich, Germany; 7 Psychiatry Neuroimaging Laboratory, Department of Psychiatry, Mass General Brigham, Harvard Medical School, Boston, Massachusetts, United States of America; 8 Graduate School of Systemic Neurosciences, Ludwig-Maximilians-Universität, Munich, Germany,; 9 International Max Planck Research School for Translational Psychiatry, Munich, Germany,; 10 German Center for Child and Adolescent Health (DZKJ), Partner site Munich, Munich, Germany,; 11 Department of Health Psychology, Germany’s State Distance-Learning University Hagen, Hagen, Germany; 12 Division of Pediatric Neuropsychology, Safe Concussion Outcome Recovery & Education Program, Children’s National Health System, Departments of Pediatrics and Psychiatry & Behavioral Sciences, George Washington University School of Medicine, Rockville, Massachusetts, United States of America; Amity University, INDIA

## Abstract

Adolescents are at an increased risk of sustaining a traumatic brain injury (TBI), which is associated with physical, cognitive, and/or emotional impairments, the so-called post-concussion symptoms (PCS). To fill the gap of German-language instruments for the age-appropriate assessment of PCS, the current study presents the translation, linguistic validation, and psychometric examination of two versions of the Postconcussion Symptom Inventory (PCSI) for adolescents (PCSI-SR13; 21 items) and their parents (PCSI-P; 20 items). Translation included iterative forward and backward translations and cognitive debriefings (CD). A total of 117 adolescents (aged 13–17 years) after TBI (3 months up to 10 years after injury) and 111 parents completed the PCSI. Both German versions were compared descriptively with the corresponding English versions. Analyses were conducted at the item and scale level. Confirmatory factor analyses (CFA) were performed, and internal consistency was examined using Cronbach’s α and McDonald’s ω. Convergent validity testing used Spearman’s ρ correlations with the Rivermead Post-Concussion Symptoms Questionnaire (RPQ). Cohen’s κ at the item level and intraclass correlation coefficients (ICC) were calculated to assess adolescent-parent agreement. The original four-factor structure could be replicated for the PCSI-SR13, but not for the PCSI-P. Internal consistency was good to excellent (≥ 0.80). Correlations (ρ ≥ 0.57) indicated a strong association with the RPQ. At the item level, the adolescent-parent agreement was fair to moderate (κ: 0.14–0.58). At the subscale level, interpretation of the ICC (ICC: 0.51–0.71) was limited due to the wide CI_95%_. In general, the psychometric properties support the applicability of the PCSI-SR13 and the PCSI-P for assessing PCS in German-speaking adolescents in the subacute and chronic phase after TBI. However, given the lack of factorial validity of the PCSI-P and the discrepancies between adolescents’ and parents’ ratings, self-report is recommended.

## Introduction

Traumatic brain injury (TBI) is internationally recognized as one of the most common traumatic causes of morbidity and mortality in adolescents [1] and is therefore of major concern for the health care system [2]. TBI can be defined as a “…consequence of force that has resulted in dysfunction and/or injury to the brain and may be associated with contusion or injury to the scalp, bony skull, vessels, brain tissue, and/or dura” [3].

Worldwide, the incidence of pediatric TBI varies widely between countries (e.g., 12 cases per 100,000 per year in Sweden, 468 cases per 100,000 per year in Australia) [2]. Road traffic accidents are reported as the leading injury cause among adolescents and young adults in European countries [4]. In 2019, 430.5 per 100,000 adolescents aged 15–17 years were treated in German hospitals [5]. Based on the *Glasgow Coma Scale* (GCS) classification [6], most commonly TBIs are classified as mild (GCS ≥ 13), accounting for up to 97.3% of all cases [7]. Overall, adolescents between the ages of 15 and 18 are most at risk for TBI [2,8], with males twice as likely as females [9].

In recent years, there has been a growing awareness that TBI should be considered not only as an acute, but also as a chronic condition with long-term consequences [10]. Similarly, the assumption that children and adolescents recover better or faster from TBI than adults due to greater neural plasticity at younger ages may be outdated [11–13], as brain functions and structures change rapidly, especially during adolescence [14]. The developing brain during adolescence is considered a major vulnerability factor for long-term complications following TBI [15,16].

Regardless of severity, TBI leads to heterogeneous impairments to the brain [17] that has been found to negatively affect children’s and adolescent’s cognitive, emotional, behavioral, and social development.

Acute or persistent symptoms following TBI are commonly subsumed under the term post-concussive symptoms (PCS). PCS include symptoms such as balance problems, blurred or double vision, headaches, concentration and/or memory difficulties, impulsive behavior [18,19], fatigue [20] and other complaints [12]. PCS are mostly observed in the context of mild TBI, although they can appear among patients after moderate or severe TBI [21]. In many cases, children and adolescents report PCS in the acute phase after TBI which mostly resolve within a few weeks [22]. However, in some individuals regardless of the TBI severity [13], symptoms remain for months [23], in a few cases even throughout the whole lifespan [18]. Based on their duration, PCS are commonly categorized as acute (lasting less than one month after TBI), subacute (persisting more than one month to 12 months after TBI), and chronic (more than one year after TBI) [24,25]. PCS that persist for more than a month are usually referred to as persistent PCS (PPCS) [18,26].

Typically, impact of P/PCS in adolescents can be assessed using self- and/or parent-report [22]. For the Anglo-American region, several instruments are available for assessing P/PCS in children and adolescents [27]. These include the Head-Brain Injury (HBI) [26], the Concussion Symptom Inventory (CSI) [28], the Post-Concussion Scale (PPCS) [29], the Rivermead Post-Concussion Questionnaire (RPQ) [30], and the Postconcussion Symptom Inventory (PCSI), based on the Postconcussion Scale [29]. The PCSI [31,32] presents notable advantages over the other mentioned measures. While both PCSI and HBI offer validated parent-report versions and are suitable for children under 12 years of age, the PCSI’s three age-appropriate versions make it a more adaptable and effective tool for assessing PCS in children and adolescents across different developmental stages [27]. Although the RPQ is similar to the PCSI in terms of wording and items, it differs in its administration. The PCSI assesses pre-injury symptoms separately using the Retrospective Adjusted Post-Injury Difference (RAPID) score, which detects clinically significant changes. In contrast, the RPQ integrates pre-injury symptoms into its response scale. Given the challenge of obtaining reliable pre-injury data in children, especially those with early childhood TBI [31], the PCSI’s two-day post-injury assessment, may be more effective for evaluating subacute and chronic PCS than the RPQ. Furthermore, the PCSI [31,32] is recommended by the Traumatic Brain Injury Outcomes Workgroup [33] for the evaluation of P/PCS in pediatric populations, underscoring its utility and validity as a tool for assessing P/PCS in both children and adolescents. The pediatric versions of the PCSI have been modified by consensus of experienced clinical pediatricians for specific age groups: PCSI-SR5 for ages 5–7, PCSI-SR8 for ages 8–12, and PCSI-SR13 for ages 13–18. Additionally, there is a parent version covering all age-groups (PCSI-P) [31]. The post-TBI version focuses on the level of symptom burden after injury. In addition, items can be grouped into four subgroups of symptom dimensions (*Physical*, *Cognition*, *Fatigue* and *Emotional*), allowing for individualized treatment planning in the clinical context [31]. By providing a parent version, information from different sources can be taken into account [31].

It is important to note that parent ratings often do not fully represent the child’s subjective perception of symptoms and may therefore reflect a different viewpoint [34]. In particular, in relation to post-concussion symptoms after TBI, agreement with parents has been found to be lower in adolescents aged 16–18 years compared to younger age groups [35,36]. Also, adolescents may report more symptoms [35] and higher symptom burden [37] compared to their parents.

To date, there is a lack of age-appropriate German instruments to assess P/PCS. Therefore, the main aim of the current study is to present the German translation, linguistic validation and psychometric characteristics of the original English versions of the PCSI in adolescents in a subacute and chronic phase after TBI and their parents and to examine their psychometric properties. As a secondary aim, we investigate the agreement between adolescents and parents in rating symptom burden to derive recommendations on whether the PCSI-P can serve as a complementary perspective to the symptom assessment provided by the PCSI-SR13.

## Materials and methods

### Data collection

#### Study sample.

Data collection was conducted as part of the multicenter project Qualitative of Life after Brain Injury in Children and Adolescents (QOLIBRI-KID/ADO) from 1th January 2019–31th January 2022 at twelve recruiting hospitals in Germany. Participants were retrospectively selected from the databases of the participating sites according to the inclusion criteria. Invitations were mailed. Those who were interested contacted the research team to enroll in the study, resulting in a self-selected purposive sample. The project enrolled children aged 8–12 years and adolescents aged 13–17 years who met the following inclusion criteria: A diagnosis of TBI (S06.-) according to the International Statistical Classification of Diseases and Related Health Problems (ICD-10) [38] obtained at least three months but no later than ten years prior to study participation, information on TBI severity according to the GCS, or, alternatively, medical records from which the severity could be inferred, and the ability to understand and complete the questionnaires. Children and adolescents with epilepsy and severe mental illness prior to TBI, severe polytrauma and/or terminal illness were excluded from the study. The only requirement for parental participation was sufficient proficiency in the German language to read and complete the questionnaires. In total, the QOLIBRI-KID/ADO study invited more than 5000 families, of whom 148 adolescents aged 13–17 years participated. Adolescents were interviewed face-to-face either online (*n* = 37) or on site at one of the recruiting clinics (*n* = 111). Parent interviews were conducted exclusively in paper-pencil format and completed by one parent per family. Due to the project’s objectives, which included the administration of neuropsychological tests, it was necessary to conduct oral interviews with the participating children and adolescents. Furthermore, it was expected that some children and adolescents would have difficulties with written assessments due to their age and/or TBI-related cognitive impairments. To ensure that data from all participating children and adolescents were assessed equally, we opted to conduct oral interviews with all of them. Any parental inquiries were clarified with the parents prior to or after the interviews with their children. Parents were instructed to complete the study questionnaires close to the interview date of their child. Written informed consent for study participation in the face-to-face-setting was obtained from both the participating adolescents and their parents at the time of data collection. In the case of online interviews, written informed consent was obtained from the participating children and adolescents, as well as their parents, prior to the interview, via postal mail.

**Fig 1:**
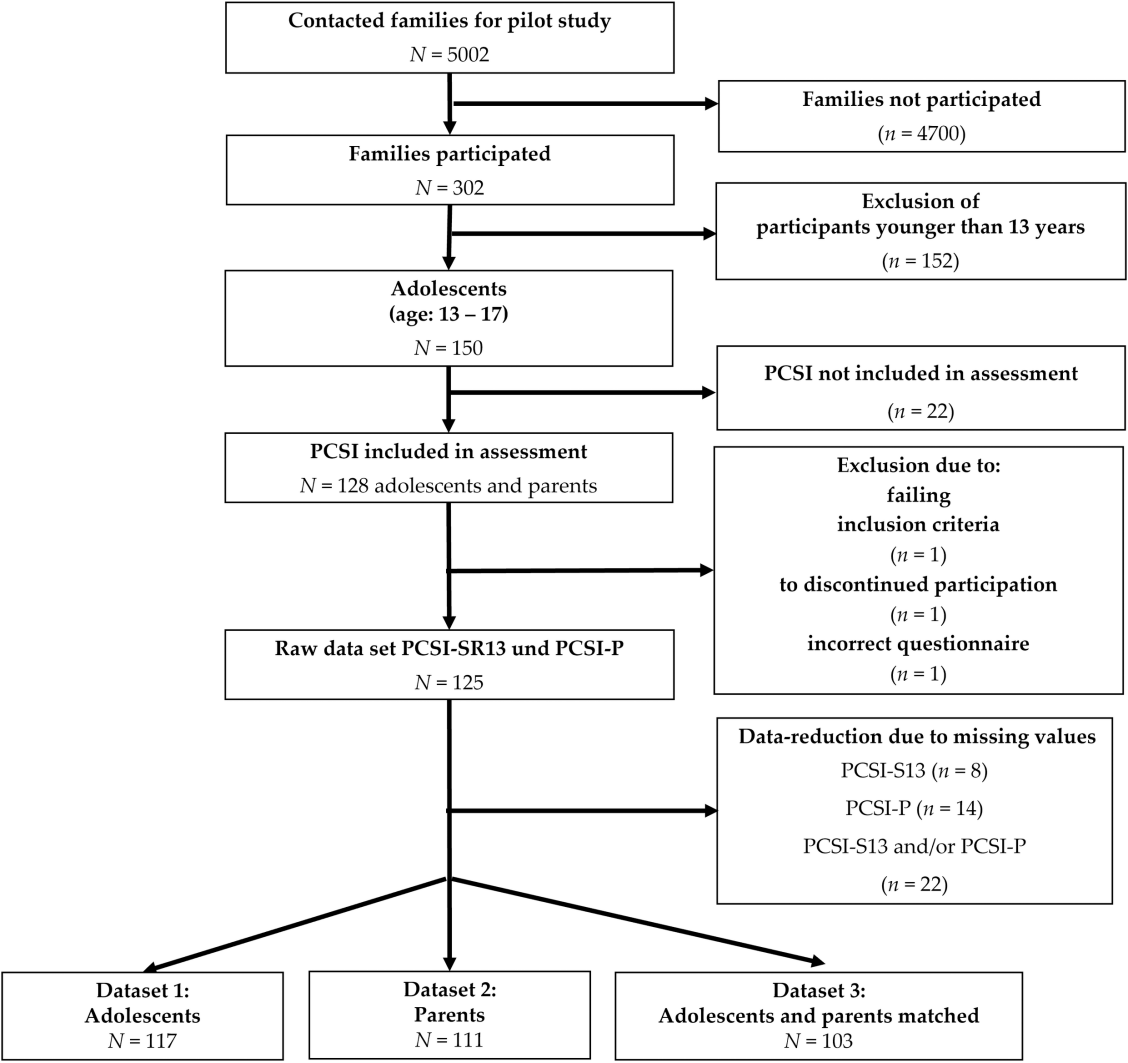
Overview of sample attrition for the current study. Data set 1 (adolescents) and 2 (parents) were used separately for descriptive and psychometrical analyses. Dataset 3 consisted of matched adolescent-parent-dyads for the agreement analyses.

This study focuses on the group of 13–17-year-old adolescents and their parents. Fig 1 provides an overview of sample attrition for the current study.

#### Ethical approval and regulations for data collection and storage.

The study was approved by the Ethics Committee of the University Medical Center Göttingen (application number: 19/4/18). In addition, the legal requirements of the Harmonised Tripartite Guideline For Good Clinical Practice (ICH-GCP) [39] published by the International Council on Harmonization’s Technical Requirements for Registration of Pharmaceuticals for Human Use as well as the Declaration of Helsinki: Ethical Principles for Medical Research Involving Human Subjects [40] released by the *World Medical Association* were considered during data collection. Data collection, storage, and processing were performed in accordance with the *European General Data Protection Regulation* (GDPR) [41].

#### Sociodemographic and injury-related data.

For adolescents, information on age, gender, TBI severity (mild, moderate, severe), time since injury, functional recovery at time of assessment, and cause and location of the accident was reported. The functional recovery of the adolescents was assessed by the examiner using the *Kings Outcome Scale for Childhood Head Injury* (KOSCHI) [42]. The KOSCHI is a clinical tool, which assesses recovery after TBI using the following categories: 1 = *dead*, 2 = *vegetative state*, 3a = *lower severe disability*, 3b = *upper severe disability*, 4a = *lower moderate disability*, 4b = *upper moderate disability*, 5a = *good recovery*, 5b = *intact recovery*. Only participants who with a score of 3 or higher were included in the study. For both parents the highest level of educational attainment and employment status were assessed. The marital status was only assessed for the parent who completed the questionnaire.

### Instruments

#### Postconcussion Symptom Inventory (PCSI-SR13 und PCSI-P).

The *Postconcussion Symptom Inventory* (PCSI) was originally developed to assess self- or parent-reported PCS after mild TBI in children and adolescents [31]. The PCSI-SR13 is applicable to adolescents (13–18 years) assessing PCS-related items after TBI [31,43]. Originally, the interpretation is based on the RAPID score, which determines clinically significant changes before and after injury [32]. In the current study, the post-TBI version was used to indicate the severity of current symptom distress and those experienced over the past two days using a 7-point Guttman scale with three anchor responses (from 0 = *Not at all a problem*, 3 = *Moderate problem*, and 6 = *Severe problem*). For the PCSI-S13, scores can be calculated for four symptom subscales: *Physical* (8 items; scores ranging from 0 to 48), *Emotional* (4 items; scores ranging from 0 to 24), *Cognition* (6 items; scores ranging from 0 to 36), and *Fatigue* (3 items; scores ranging from 0 to 18). A score for the total scale can be calculated based on the values of all items (21 items; scores ranging from 0 to 126).

The PCSI-P can be used to assess PCS of the affected adolescents rated by their parents. The parent version consists of 20 items, as the item *Feeling slowed down* used in the PCSI-SR13 version is omitted. Parents are asked to rate their children’s symptoms burden over the past two days using the 7-point Guttman scale mentioned above. Also, for the PCSI-P, scores can be calculated for four symptom subscales: *Physical* (8 items; scores ranging from 0 to 48), *Emotional* (4 items; scores ranging from 0 to 24), *Cognition* (5 items; scores ranging from 0 to 30), and *Fatigue* (3 items; scores ranging from 0 to 18). The score for the total scale using 20 items ranges from 0 to 120.

For the post versions of the PCSI-SR13 and PCSI-P, higher (sub-) total scale scores indicate higher levels of PCS burden.

Satisfactory values for internal consistency have been reported for both versions (PCSI-SR13: total scale α = 0.94, subscales α = 0.79 to α = 0.93; PCSI-P: total scale α = 0.94, subscales α = 0.83 to α = 0.92) [31]. For convergent validity, statistically significant high positive correlations with a previously validated checklist have been obtained: PCSI-SR13 (18 items) *r*_s_ = 0.86, *p* < .001 and PCSI-P (17 items) *r*_s _= 0.93, *p* < .001 [31]. These values were used to evaluate the German validation.

#### Rivermead Post-Concussion Symptoms Questionnaire (RPQ).

The *Rivermead Post-Concussion Symptoms Questionnaire* (RPQ) [30] was originally developed to assess PCS after mild TBI in English-speaking individuals 16 years of age and older. RPQ includes 16 items rated on a 5-point Likert-type scale (0 = no experienced at all, 1 = no more of a problem (than before), 2 = a mild problem, 3 = a moderate problem, 4 = a severe problem). For score calculation, response option 1 (no more of a problem) should be treated as 0. The total scale sum score ranges from 0 (*no increased difficulties since TBI*) to 64 (*most severe symptoms*) with higher values meaning greater symptom burden. Apart from the score for the total scale, scores for three subscales can be derived: the *Somatic* scale (9 items; from 0 to 36), the *Emotional* scale (4 items; from 0 to 16), and the *Cognition* scale (3 items; from 0 to 12.) Overall, good psychometric characteristics have been reported in adults post TBI [30]. Validated German versions for adolescents (aged 13–17 years) in a subacute and chronic phase after TBI and their proxies are available [44]. The authors report good internal consistency for the assessment in self- and parent-report (Cronbach’s α: 0.81–0.91 and McDonald’s ω: 0.84–0.95) [44].

### Statistical analyses

Most analyses were performed using IBM SPPS Software Package (version 28.0) [45]. For factor analyses, R version 4.3.0 [46] and the *lavaan* package [47] were used. For each questionnaire, only complete cases were included in the analyses. If not otherwise specified, the significance level was set at 5% for all analyses.

#### Missing values.

Analyses on item level used data solely for adolescents and parents for whom questionnaires were available for the respective analyses (*N*_PCSI-SR13_* *= 117, 6.4% missing datasets; *N*_PCSI-P _= 111, 11.2% missing datasets; *N*_PCSI-SR13 and PCSI-P _= 103, 17.6% missing datasets). Missing subscale values were replaced by the median of the respective subscale to preserve the information for statistical analyses, if not more than one item was missing on subscale level or not more than three items were missing for the total scale (*N*_*PCSI-P and RPQ*_* = *122, 2.4% missing datasets; *N*_*PCSI-SR13 and PCSI-P *_= 115, 8.0% missing datasets).

#### Descriptive statistics.

For adolescents’ sociodemographic data (age and gender) mean (*M*), standard deviation (*SD*), minimum (*Min*) and maximum (*Max*) were reported. Absolute and relative frequencies were reported for adolescents’ injury-related (TBI severity, time since injury, cause of injury, accident location, functional recovery) and parents’ sociodemographic characteristics (highest level of educational attainment, employment status, family situation of the parent who completed the questionnaire).

#### Descriptive comparison of response patterns.

The current sample of adolescents and parents was compared with the respective samples used in the English validation study by Sady et al. [31]. Differences were reported descriptively at the item level for adolescents and their parents. Items with *M* differences being equal to or less than half the *SD* reported for the samples of the original English study were considered to be nearly comparable [48,49].

To display the distributions of the response categories, the 7-point Guttman scale was trichotomized, corresponding to the presentation in the validation study by Sady et al. [31]. The results of the descriptive comparison between the samples of the current study and the original English study were presented at the subscale level.

#### Item characteristics.

In all summaries, absolute and relative frequencies, *M*, *SD*, range, skewness (*SK*) and kurtosis (*KU*) were reported for each item. In accordance with Bulmer [50], values for *SK* and *KU* between −2 and +2 were considered acceptable. Response behavior at the item level was analyzed for floor and ceiling effects with respect to the relative frequencies of the lowest (0 = “not a problem”) and the highest (6 = “severe problem”) categories of the 7-point Guttman scale. The cut-off for both was defined by ≤ 15% indicating no ceiling or floor effects for the respective item [51]. For the subscales and for the total scale, the following descriptive statistics were presented for both the PCSI and the RPQ in self- and parent-report: *M*, *SD*, median (*Mdn*) and the values of the Shapiro-Wilk normality tests (*W*). A test *p*-value below 0.05 indicated a significant deviation from the normal distribution [52], suggesting the use of non-parametric tests.

#### Factorial validity.

The validity of the proposed four-factor structure comprising the *Physical*, *Cognition*, *Emotional*, and *Fatigue* subscales was tested in two ways. First, a confirmatory factor analysis (CFA) for metric data with maximum likelihood estimator (ML) was performed to compare the results of the present study with those reported by Sady et al. [31]. Secondly, a CFA appropriate for ordinal data with robust weighted least squares estimator (WLSMV) was performed to account for the ordinal nature of the response scale. Model fit interpretation for both methods were based on the following goodness-of-fit indices: ratio of chi-square to degrees of freedom (χ2/*df*; ≤ 2 = good fit), *Comparative Fit Index* (CFI ≥ 0.95), *Tucker-Lewis-Index* (TLI ≥ 0.95), *Standardized Root Mean Square Residual* (SRMR ≤ 0.08), *Root Mean Square Error of Approximation* with a confidence interval ([CI]) of 90% (RMSEA ([CI_90%_]): 0.10 = adequate fit, 0.05 = good fit) [53–55].

#### Reliability.

To examine the reliability of the subscales and the total scale, Cronbach’s α and McDonald’s ω were calculated, with α values between 0.70 and 0.85 indicating good to excellent [56] and ω values equal or higher than 0.80 indicating good internal consistency [57]. In addition, for all subscales **Cronbach’s* α *if item omitted** was calculated. The change in reliability if an item omitted should not exceed the initial α of the scale, as this would indicate an improvement in internal consistency. To evaluate the association of the individual items with the respective sub- and total scales, corrected item-total correlations (CITC) were calculated, with a CITC ≥ 0.30 considered acceptable [58] and CITC ≥ 0.40 considered good. [59]. Furthermore, correlations between the scales were evaluated with |ρ| = 0.10 considered weak, |ρ| = 0.30 moderate and |ρ| ≥ 0.50 strong according to the respective effect sizes [58].

#### Convergent validity.

Convergent validity was tested by Spearman correlation (ρ) of the total scale and the subscales of the German versions of the PCSI-SR13/ PCSI-P and the RPQ. Correlation coefficients ρ ≥ 0.30 were considered acceptable. Strong positive correlation (ρ ≥ 0.50) between the German versions of the PCSI and the corresponding version of the RPQ was expected.

#### Agreement between adolescents and parents.

All agreement analyses were calculated with matched adolescent-parent-dyads, resulting in *N* = 103. On item level, Cohen’s weighted κ (κ_w_) was calculated. Values greater than 0.80 indicate almost perfect, values between 0.61 and 0.80 indicate substantial, values between 0.41 and 0.60 indicate moderate, values between 0.21 and 0.40 indicate fair, values between 0.01 and 0.20 indicate slight, values below 0.00 indicate insufficient agreement [60].

The unadjusted intraclass correlation (ICC) obtained from the two-way mixed-effects model with absolute agreement was used for the subscales, with values less than 0.30 indicating poor agreement, values between 0.30 and 0.50 indicating moderate agreement, and ICC greater than 0.50 indicating good agreement [61]. To evaluate whether parents of the current study tended to over-, underestimate, or agree with their children’s rating of symptom burden, differences between the absolute subscale and total scales of the PCSI-SR13 and PCSI-P were calculated. The results were grouped to three categories: Values of 0 indicated that the parental rating of symptom burden was consistent with the rating of the adolescent (category 0). Negative values indicated an overestimation (category 1) and positive values an underestimation (category 2) of symptom burden by parents [62]. The relative proportions of the categories were graphically presented in bar charts. For all analyses Item 18 (*Feeling slowed down*) of the PCSI-SR13 was excluded, since no comparable item was available in the parent version.

It was expected that agreement between adolescents and their parents would be fair to moderate.

## Results

### Translation process of the PCSI-SR13 and the PCSI-P

**Fig 2:**
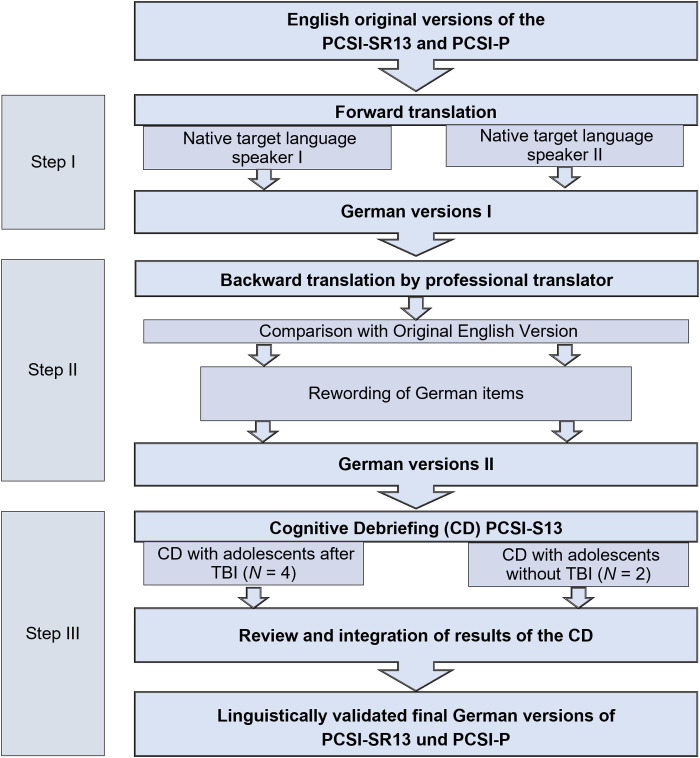
Translation and linguistic validation process. *N* = absolute number of participants.

Prior to the psychometric investigation, a translation and linguistic validation according to common guidelines [63] was conducted, which included cognitive debriefings. The translation process followed the stepwise approach for outcome instruments as recommended by von Steinbuechel et al. [64]. Fig 2 provides an overview of the single steps of this process.

In a first step, two members of the Institute of Medical Psychology and Medical Sociology (IMP/IMS) at the University Medical Center Göttingen (UMG) independently translated the English versions of the PCSI-SR13 and the PCSI-P into German. These translations were harmonized in collaboration with another member of the IMP/IMS.

In a second step, a professional translator re-translated the first harmonized German versions into English. The re-translated English versions were then checked against the original versions by three IMP/IMS staff members. Where necessary and possible, the translation of the German items was brought closer to the English ones. Deviations in the wording of individual items, where an adequate literal translation was either not possible or unusual, were clarified with one of the leading developers of the original versions of the PCSI.

In a third step, two IMP/IMS staff members conducted cognitive debriefings (CD) with a total of six adolescents aged between 14 and 15 years (two males and two females after TBI and one male and one female without a history of TBI). The CDs were conducted as a structured interview: the participants were asked to evaluate the content of the instruction, the items, and the response options regarding comprehensibility, difficulty, and relevance. Additionally, they were asked to reproduce the items in their own words and to make suggestions for changes in wording, if necessary. During the interview, the examiners recorded all answers given by the participants on a template.

After the CDs were completed, they were evaluated by three IMP/IMS staff members. After internal discussions, no further adjustments were made.

### Study sample

Overall, complete PCSI-SR13 were available for 117 (57.3% males) adolescents (age: *M* = 14.82 years, *SD* = 1.41, *Min*–*Max* = 13.00–17.11 years). Most of the adolescents (77.8%) were diagnosed with mild TBI and 56.4% had experienced a TBI more than four years before study enrollment (*M* = 4.85 years, *SD* = 2.85, *Min–Max* = 0.42–10.17 years). The most common reported cause of accident was a fall (60.7%), and most accidents occurred during sports or leisure time (about 61.5%). Based on the KOSCHI classification, the majority of the study adolescents (89.7%) were classified as having good functional recovery (KOSCHI score 5a or 5b). For more details, see Table 1.

**Table 1:** Injury and clinical related characteristics of adolescents.

Variable	Group/ Values	*N* = 117 (100%)
Gender	Female	49 (41.9%)
	Male	67 (57.3%)
	Diverse	1 (0.9%)
Age (in years)	*M* (*SD*)	14.82 (1.41)
	*Min* – *Max*	13.00–17.11
TBI severity	Mild	91 (77.8%)
	Moderate	8 (6.8%)
	Severe	18 (15.4%)
Time since injury (in years) ^a^	*M (SD)*	4.85 (2.85)
	*Min – Max*	0.42–10.17
Time since injury (in groups)	<1 years	7 (6.0%)
	1 – < 2 years	18 (15.4%)
Time since injury (in groups)	2 – < 4 years	25 (21.4%)
	4–10 years	69 (56.4%)
	Missing Values	1 (0.9%)
Cause of accident	Road traffic accident	16 (13.7%)
	Fall	71 (60.7%)
	Violence	2 (1.7%)
Cause of accident	Collision	20 (17.1%)
	Other	6 (5.1%)
	Missing Values	2 (1.7%)
Accident Location	Sports and leisure time	72 (61.5%)
	Domestic accident/ accident in domestic environment	16 (13.7%)
Accident Location	Care-/ Educational Institution	12 (10.3%)
	Other	15 (12.8%)
	Missing Values	2 (1.7%)
KOSCHI	4a “lower moderate disability”	3 (2.6%)
	4b “upper moderate disability”	9 (7.7%)
	5a “good recovery”	19 (16.2%)
	5b “intact recovery”	87 (73.5%)

*N* = absolute frequencies, % = relative frequencies, *M* = mean, *SD* = standard deviation, Min = minimum, Max = maximum. KOSCHI = Kings Outcome Scale for Childhood Head Injury.

^a^*n* = 116.

Complete PCSI-P were available for 111 parents. Most parents had a university degree (mothers: 48.6%; fathers: 55.0%). For more details, see Table 2.

**Table 2:** Sociodemographic characteristics of the parents (*N* = 111).

		Mother	Father
Variable	Category	*N = *111	
Highest level of educational attainment	None	0 (0.0%)	1 (0.9%)
	Secondary/ High School	30 (27.0%)	27 (24.3%)
	Post High School Training	19 (17.1%)	17 (15.3%)
	University	54 (48.6%)	61 (55.0%)
Highest level of educational attainment	Missing	8 (7.2%)	5 (4.5%)
Employment status	Employed (<35h/week)	38 (34.2%)	86 (77.5%)
	Employed (≥ 35h/week)	55 (49.5%)	14 (12.6%)
	Employed, but currently on sick/maternity/parental leave	1 (0.9%)	2 (1.8%)
	Housewife/houseman	9 (8.1%)	0 (0.0%)
	Job seeking/unemployed	2 (1.8%)	1 (0.9%)
	Retired	0 (0.0%)	1 (0.9%)
	Other	0 (0.0%)	3 (2.7%)
	Missing	6 (5.4%)	4 (3.6%)
Marital status ^a^	Living in a partnership	94 (84.7%)	
	Single parent	16 (14.4%)	
	Missing	1 (0.9%)	

*N* = absolute frequencies, % = relative frequencies.

^a^The marital status was assessed only for the parent who completed the questionnaire.

### Descriptive comparison of response patterns

Overall, the item characteristics of the German PCSI-SR13 were comparable to those of the original English study. However, some differences of more than-half a *SD* were observed in the items *Headache*, *Answer questions more slowly*, *Irritability* and *Sadness*.

**Fig 3:**
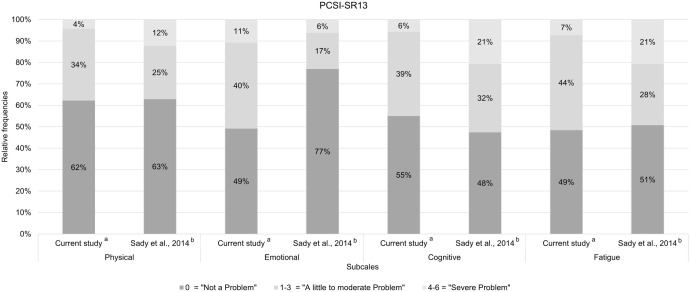
Comparison of relative frequencies of responses for the adolescents per subscale. Due to rounding, not all percentages sum up to 100%. ^a^Sample size *N* = 117. ^b^ Values from the original English study [31] summed on the subscale level. Sample size *N* = 227.

The comparison of the distribution of relative frequencies across the response categories between the German and English samples of adolescents for the *Physical*, *Cognition* and *Fatigue* subscales indicated a more heterogeneous response behavior in the adolescents of the original English study. In contrast, for the *Emotional* subscale the comparison implied a more heterogeneous response behavior in adolescents of the current study compared to those of the original English study. For more details see Fig 3.

**Fig 4:**
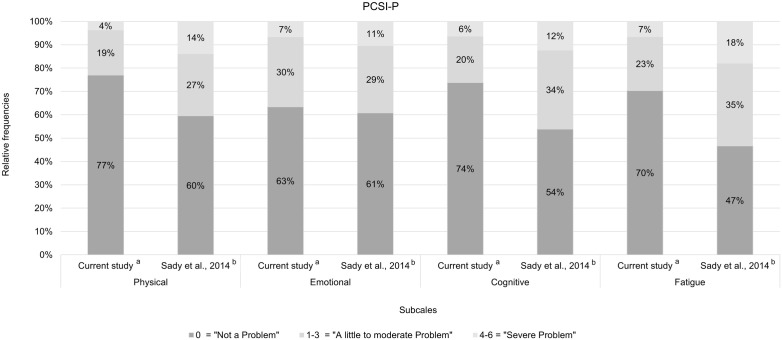
Comparison of relative frequencies of responses for the parents per subscale. ^a^ Sample size N = 111. ^b^ Values from the original English study [31] and summed on the subscale level. Sample size N = 521.

For the PCSI-P, the item mean differences between the two studies were rather small (≤ 0.5 *SD* reported for the original English study), except for the items *Headache* and *Feeling foggy*. For all subscales the comparison of the relative frequencies across response categories at the subscale level showed a more heterogeneous response pattern among parents in the original English study (see Fig 4).

### Item characteristics

The overall average item characteristics were the following: *M* = 0.92, *SD* = 0.33, *SK* = 1.71, and *KU* = 2.89 (PCSI-SR13) and *M* = 0.64, *SD* = 0.29, *SK* = 2.47, *KU* = 7.02 (PCSI-P). Both, PCSI-SR13 and the PCSI-P items were skewed to the right. For more details on item characteristics, see Table A1 in S1 Appendix. For each item of the German PCSI-SR13 and PCSI-P, the distribution of relative frequencies of the responses on the 7-point Guttman scale is provided in Table A2 in in S2 Appendix. For all items floor but no ceiling effects were found. Table A3 in in S3 Appendix provides a comparison of the main item characteristics of the PCSI-SR13 and the PCSI-P from the current study with those from the English validation study [31].

Table 3 provides an overview on descriptive statistics of the sub- and total scales of the PCSI and the RPQ for self- and parent-report. For all scales, the Shapiro-Wilk test was significant, indicating that the data were not normally distributed. Overall, mean values indicated a rather low symptom burden for the adolescents of the German study.

**Table 3:** Descriptive statistics of the sub- and total scales for the PCSI and the RPQ.

Instrument	Scales (range)	Self-report (*N *= 122)				Scales (range)	Parent-report *(N* = 115)			
		*M*	*SD*	*Mdn*	*W*		*M*	*SD*	*Mdn*	*W*
**PCSI**	Physical (0-48)	6.10	6.68	4	**0.81**	Physical (0-48)	3.82	5.96	2	**0.65**
	Emotional (0-24)	5.02	5.53	3	**0.83**	Emotional (0-24)	3.26	4.83	1	**0.72**
	Cognition (0-36)	5.43	5.34	4	**0.84**	Cognition (0-30)	3.19	4.78	1	**0.72**
	Fatigue (0-18)	3.21	3.52	2	**0.82**	Fatigue (0-18)	2.04	3.50	0	**0.65**
	Total scale (0-126)	19.75	17.88	14	**0.86**	Total scale (0-120)	12.30	15.66	7	**0.76**
**RPQ**	Somatic (0-36)	3.50	4.78	2	**0.76**	Somatic (0-36)	2.80	4.87	0	**0.65**
	Emotional (0-16)	2.32	3.58	0	**0.70**	Emotional (0-16)	1.58	3.10	0	**0.58**
	Cognition (0-12)	2.00	2.78	0	**0.73**	Cognition (0-12)	1.94	2.99	0	**0.70**
	Total scale (0-64)	7.79	9.52	4	**0.80**	Total scale (0-64)	6.32	8.94	2	**0.75**

*N* = absolute frequencies, *M* = mean, *SD* = standard deviation, *Mdn* = median, *W* = value of Shapiro-Wilk normality test, PCSI = Post-Concussion Symptom Inventory (self-report: PCSI-SR13; parent-report: PCSI-P), RPQ = Rivermead Post-Concussion Symptoms Questionnaire. *W*-values in bold indicate significant deviation from the assumption of normally distributed data.

### Factorial validity

Following the approach of the original English validation study, CFA for metric data were estimated for the PCSI-SR13 and for the PCSI-P. While the factorial structure could be replicated for the four-factor model of the PCSI-SR13 with nearly comparable indices reported for the English version, none of the specified cutoffs were met for the PCSI-P (see Table 4).

**Table 4:** Fit indices obtained from the CFA with Maximum-Likelihood estimation.

Questionnaire	Sample	χ^2^	*df*	χ^2^/*df*	*p*	CFI	TLI	RMSEA	CI_90%_	SRMR
PCSI-SR13	Current Study (*N *= 117)	387.82	183	2.12	*<0.001*	0.85	0.83	**0.10**	[**0.08** – 0.11]	**0.08**
	Sady et al ^a^ (*N *= 227)	n.a	n.a.	2.66	n.a.	0.90	n.a.	**0.09**	n.a.	**0.06**
PCSI-P	Current Study (*N *= 111)	576.26	164	2.51	*<0.001*	0.78	0.75	0.15	[0.14 – 0.16]	0.11
	Sady et al ^a^ (*N* = 521)	n.a.	n.a.	5.63	n.a.	0.89	n.a.	**0.09**	n.a.	**0.05**

χ^2^ = Chi-Square **df* *= degrees of freedom, χ^2^/*df* = ratio chi-square to degrees of freedom (cutoff: ≤ 2), *p* = p-value, CFI = Comparative Fit Index (Cutoff: ≥ 0.95), TLI = Tucker-Lewis-Index (cutoff: ≥ 0.95), RMSEA = Root Mean Square Error of Approximation (cutoff: excellent ≤ 0.05, moderate ≤ 0.10) with 90% confidence interval [CI], SRMR = Standardized Root Mean Square, cutoff: ≤ 0.08), PCSI = Post-Concussion Symptom Inventory (self-report: PCSI-SR13; parent-report: PCSI-P), *N* = absolute frequencies. Bold values indicate at least satisfactory model fit according to the indicated cutoffs, values in italics are significant at 5%.

^a^Values were taken from the original English study by Sady et al. [31]. n.a. = values were not reported.

The four-factor model using the 7-point Guttman scale and the WLMSV estimator could not be replicated for either the PCSI-SR13 or the PCSI-P. This can be explained by the small sample size of adolescents and parents in relation to the number of parameters to be estimated. Also, the previous analyses demonstrated a partly pronounced right-skewed distribution of the data, especially for the parents. Therefore, the original response scale for the PCSI-SR13 was trichotomized, i.e., reduced to three response categories, following the 3-point response scale of the PCSI for children between the ages of 5 and 7 (for more details, see Sady et al. [31]). While the response category 0 was retained, response categories 1–3 and response categories 4–6 were each aggregated to one response category (1 and 2, respectively). Four of the fit indices indicated at least acceptable fit with χ2(183) = 263.82, *p* < 0.001, χ2/*df* = 1.44, CFI = 0.96, TLI = 0.96, RMSEA [CI90%] = 0.06 [0.04, 0.08], SRMR = 0.10.

Since the descriptive analysis on the item level revealed that the relative frequencies for the response category 0 (“not a problem”) were always at least up 50% for the PCSI-P, the response scale was dichotomized (0 = 0 and 1–6 = 1) and the model was re-estimated. However, no valid model could be identified for the PCSI-P.

### Reliability

The internal consistency of the four subscales and the total scale of the PCSI-SR13 and the PCSI-P were at least good for both, Cronbach’s α and McDonald’s ω (see Table 5). For most subscales, Cronbach’s α values were comparable or even higher for the German versions of the PCSI-SR13 and the PCSI-P compared to the respective English versions. Cronbach’s α of the *Cognition* subscale of the German version of the PCSI-SR13 was substantially lower compared to the value reported for the English version (α = 0.83 vs. α = 0.93). However, the value was above the predefined cut-off, indicating high internal consistency. For the PCSI-SR13, omitting the item *Visual problems (double vision, blurring)* from the *Physical* subscale and the item *Sleep more than usual* from the *Fatigue* subscale led to an increase, respectively (from α = 0.84 to α = 0.85 and from α = 0.84 to α = 0.88). For the PCSI-P, Cronbach’s α increased when omitting the item *Headache* from the *Physical* subscale and the item *Feeling “foggy”* from the *Cognition* subscale (from α = 0.88 to α = 0.89 and from α = 0.89 to α = 0.90). However, changes in Cronbach’s α were rather negligible (max. Δ0.04). The correlations of the items with the respective scale were at least satisfactory for both the PCSI-SR13 and the PCSI-P. For further information see Table 5.

**Table 5:** Reliability coefficients for the PCSI-SR13 and the PCSI-P.

Questionnaire	Scale	Cronbach’s α	Cronbach’s α If Item Is Omitted	McDonald’s ω	CITC range	Cronbach’s α Sady et al^a^
PCSI-SR13 (*N* = 117)	Physical	**0.84**	0.80–0.85	**0.84**	0.35–0.76	**0.86**
	Emotional	**0.87**	0.80–0.87	**0.88**	0.65–0.81	**0.85**
	Cognition	**0.83**	0.78–0.82	**0.83**	0.46–0.69	**0.93**
	Fatigue	**0.84**	0.67–0.88	**0.86**	0.59–0.80	**0.79**
	Total scale	**0.93**	0.93–0.93	**0.93**	0.28–0.76	**0.94**
PCSI-P (*N* = 111)	Physical	**0.88**	0.84–0.89	**0.87**	0.45–0.80	**0.86**
	Emotional	**0.90**	0.84–0.89	**0.90**	0.71–0.85	**0.83**
	Cognition	**0.89**	0.83–0.90	**0.91**	0.57–0.85	**0.92**
	Fatigue	**0.92**	0.84–0.91	**0.92**	0.80–0.90	**0.87**
	Total scale	**0.95**	0.94–0.95	**0.95**	0.55–0.78	**0.94**

CITC = Corrected item-total correlation, PCSI = Post-Concussion Symptom Inventory (self-report: PCSI-SR13; parent-report: PCSI-P), *N* = absolute frequencies. Cronbach’s α und McDonald’s ω values in bold indicate at least a good scale reliability (α ≥ 0.70 or. ω ≥ 0.80).

^a^Values were taken from the original English study [31]. *N*_PCSI- SR13 _= 223, *N*_PCSI-P_ = 521.

As shown in Table 6, for the German versions of the PCSI-SR13 and PCSI-P and the corresponding English versions, the correlations between the subscales were at least moderate (|ρ| ≥ 0.30).

**Table 6:** Correlations between subscales for the PCSI-13 and the PCSI-P.

Questionnaire	Scale	Current study				Sady et al.^a^			
		(1)	(2)	(3)	(4)	(1)	(2)	(3)	(4)
PCSI-SR13 (*N* = 117)	Physical (1)	1	—	—	—	1	—	—	—
	Emotional (2)	**0.61**	1	—	—	**0.52**	1	—	—
	Cognition (3)	**0.61**	**0.69**	1	—	**0.73**	0.45	1	—
	Fatigue (4)	**0.64**	**0.59**	**0.62**	1	**0.65**	0.44	**0.69**	1
	Total scale	**0.84**	**0.84**	**0.87**	**0.80**	n.a.	n.a.	n.a.	n.a.
PCSI-P (*N* = 111)		(1)	(2)	(3)	(4)	(1)	(2)	(3)	(4)
	Physical (1)	1	—	—	—	1	—	—	—
	Emotional (2)	**0.61**	1	—	—	**0.63**	1	—	—
	Cognition (3)	**0.51**	**0.63**	1	—	**0.72**	**0.60**	1	—
	Fatigue (4)	**0.61**	**0.56**	**0.52**	1	**0.69**	**0.54**	**0.68**	1
	Total scale	**0.84**	**0.84**	**0.76**	**0.77**	n.a.	n.a.	n.a.	n.a.

PCSI = Post-Concussion Symptom Inventory (self-report: PCSI-SR13; parent-report: PCSI-P), *N* = absolute frequencies. Values in bold indicate a strong correlation coefficient (|ρ| ≥ 0.50).

^a^Values were taken from the original English study by Sady et al. [31]. *N*_PCSI- SR13_ = 223, *N*_PCSI-P_ = 521. n.a. = values were not reported.

### Convergent validity

The subscale and total scale correlations between the PCSI and RPQ were strong (ρ > 0.5) for both self-report and parent report (see Table 7).

**Table 7:** Correlations between the PCSI and the RPQ, separated for self- and parent report.

Origin	*N*	Total Scale (PCSI, RPQ)	Emotional (PCSI, RPQ)	Cognition (PCSI, RPQ)	Physical (PCSI) Somatic (RPQ)
Self-report	122	**0.79**	**0.72**	**0.68**	**0.70**
Parent-report	115	**0.76**	**0.65**	**0.66**	**0.53**

*N* = absolute frequencies, PCSI = Post-Concussion-Symptom-Inventory (Values in bold indicate strong correlation-coefficients (|ρ| ≥ 0.5).

### Agreement between Self-report (PCSI-SR13) and Parent-report (PCSI-P)

Fair to moderate agreement (κ_w_ = 0.21 to 0.60) was found for most items. Slight agreement (κ_w_ = 0.00 to 0.20) was observed for the items *Nausea* and *Feeling “foggy”* (see Table 8).

**Table 8:** Squared weighted Cohen’s k values for the agreement between adolescents and parents on item level.

Scale	Item	k_w_	SE	CI_95%_	*p*
Physical	Headache	**0.58**	0.09	0.40–0.76	< 0.001
	Nausea	0.18	0.11	−0.03–0.40	0.050
	Balance Problems	**0.47**	0.15	0.17–0.76	< 0.001
	Dizziness	**0.51**	0.09	0.34–0.69	< 0.001
	Visual Problems (double vision, blurring)	0.21	0.10	0.02–0.40	0.011
	Move in a clumsy manner	0.27	0.08	0.10–0.43	0.005
	Sensitivity to light	**0.45**	0.12	0.21–0.69	< 0.001
	Sensitivity to noise	0.39	0.09	0.20–0.57	< 0.001
Emotional	Irritability	**0.48**	0.10	0.28–0.67	< 0.001
	Sadness	**0.48**	0.10	0.28–0.69	< 0.001
	Nervousness	0.32	0.10	0.12–0.53	< 0.001
	Feeling more emotional	**0.44**	0.11	0.24–0.65	< 0.001
Cognition	Feeling “foggy”	0.14	0.10	−0.06–0.34	0.116
	Difficulty concentrating	0.32	0.11	0.11–0.54	< 0.001
	Difficulty remembering	0.31	0.12	0.08–0.54	< 0.001
	Get confused with directions or tasks	0.32	0.11	0.10–0.55	< 0.001
	Answers questions more slowly than usual	0.37	0.11	0.16–0.58	< 0.001
Fatigue	Fatigue	0.34	0.10	0.15–0.54	< 0.001
	Drowsiness	**0.46**	0.10	0.26–0.66	< 0.001
	Sleep more than usual	0.40	0.13	0.15–0.65	< 0.001

Number of observations = 103. kw = weighted Cohen’s k, SE = standard error, [CI_95%_] = Asymptotic confidence interval, p = level of significance. Values highlighted in bold indicate at least moderate agreement (kw > 0.41).

At the subscale level and for the total scale, agreement between adolescents and their parents was generally good (unadjusted ICC > 0.50) (see Table 9). However, the wide ranges of the CI_95%_ indicated that the scale means should be interpreted with caution. This particularly applies the *Cognition* subscale, as the lower CI was below the cut-off of 0.30.

**Table 9:** Unadjusted intraclass-correlation for the agreement between adolescents and parents on subscale level and for the total scale.

Scale	Unadjusted ICC	CI_95%_
Physical	**0.63**	0.44–0.75
Emotional	**0.71**	0.57–0.80
Cognition ^a^	**0.51**	0.28–0.65
Fatigue	**0.63**	0.45–0.75
Total scale	**0.67**	0.50–0.78

Number of observations = 103. ICC = intraclass correlation coefficient with a 95% confidence interval, [CI_95%_]. Values highlighted in bold are significant on the 0.001 level. ICC-Values > 0.30 indicate at least moderate agreement.

^a^Cognition subscale: Adolescents’ subscale score without item 18.

**Fig 5:**
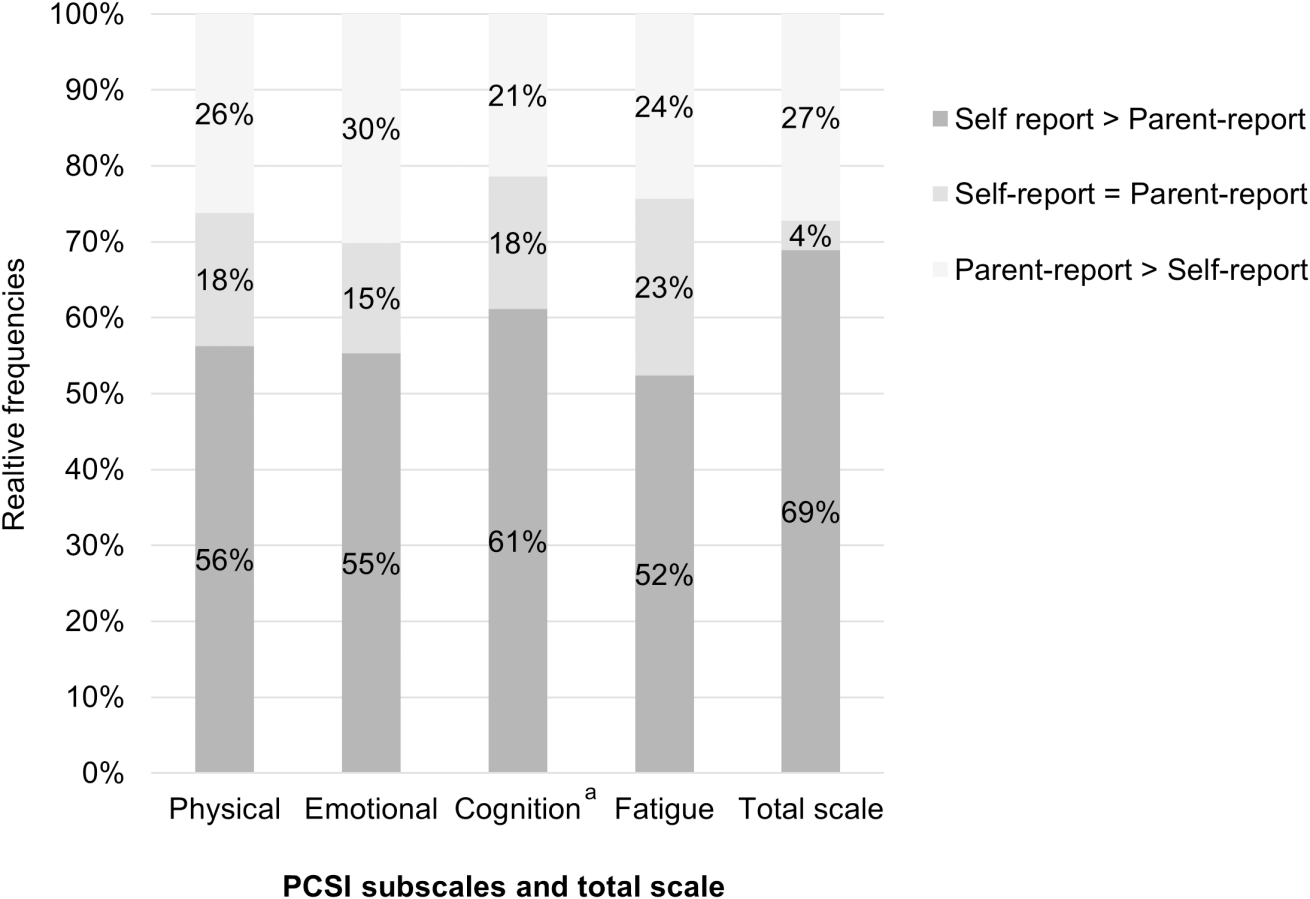
Comparison (in %) of absolute subscale and total scale values between self-report (PCSI-S13) and parent-report (PCSI-P). Number of observations = 103. “Self-report > Parent-report” = proportion of higher scale scores for adolescents compared to parents, “Self-report = Parent-report” = proportion of equal scale scores between parents and adolescents, “Parent-report > Self-report” = proportion of higher scale scores for parents compared to adolescents. ^a^ Cognition subscale: Adolescents’ subscale score without item 18.

Fig 5 shows the relative frequencies of agreement and differences in self and parent response behavior at the subscale level and for the total scale. Adolescents were more likely to rate symptom burden higher than their parents on both the subscales and the total scale. At the subscale level, this was particularly evident for the *Cognition* subscale (approx. 61% higher ratings by adolescents). The lowest proportion of agreement between adolescents and parents was found for the *Emotional* subscale (approx. 15%). In addition, compared to the other subscales, the Emotional subscale also had the highest proportion of parents reporting higher symptom burden than their children (approx. 30%).

## Discussion

Given the lack of age-appropriate German-language instruments assessing PCS after pediatric TBI, the aim of the current study was to translate, linguistically validate, and investigate the psychometric properties of the German versions of the PCSI-SR13 (for adolescents aged 13–17 years) and the PCSI-P (parent version). For this purpose, the reliability and validity of both versions of the PCSI were examined and the results were compared with those of the original English validation study. In order to investigate whether the parental report can be used as a complementary source of information on P/PCS, the agreement between the adolescents and their parents’ ratings were determined. Overall, the internal consistency and the convergent validity with the RPQ were found to be good.

PCS commonly resolve after two weeks [65] and predominantly may last up to six months [15]. In our study, mainly individuals in a subacute and chronic TBI phase participated, in which however PCS may still persist [25]. By conducting a descriptive comparison between the findings in the acute TBI sample of the original English study [31] and our sample, we were able to identify potential similarities and differences in symptoms between acute and subacute/chronic samples. Based on the results of psychometric analyses and these comparisons, we can recommend the applicability of the PCSI for the assessment of P/PCS in clinical practice. However, the four-factor structure of the PCSI-P could not be replicated. In addition, item-level agreement between adolescents and their parents regarding symptom burden was rather low. In conclusion, self-report assessment of P/PCS should be preferred to parental ratings in adolescents in a subacute and chronic TBI phase.

The descriptive comparison with the original English study revealed that on average symptom burden in both studies was rated to be rather low, both in self- and parent-reports. However, descriptive analyses also revealed that more variety in the severity of reported symptom burden was found in the original English samples. Although most of the adolescents in the current study had suffered a TBI more than 4 years prior to enrollment, the results can be considered comparable to those of the original English validation study in terms of overall symptom burden. On the one hand, this may be due to the fact, that TBI is a heterogeneous condition [17] that is not only acute, but it can develop into a potentially chronic state [10] for which it is not always possible to predict how it will affect an individual adolescent in the short or long term. On the other hand, developmental changes during adolescence should also be considered. These changes can induce post-concussion-like symptoms in the general pediatric population independent of a TBI history [66]. These findings emphasize the need to follow the development of adolescents after a TBI with age-appropriate instruments beyond the acute phase of injury. Additionally, it can be suggested that, in order to gain a more comprehensive understanding of an individual’s current symptomatology, pre-injury symptom reports should also be taken into account.

In the current study, symptom burden was rated relatively low on average by adolescents and especially by their parents. This is reflected in substantially right-skewed item scores, showing floor effects. This result was expected, as most adolescents in the current sample had experienced a mild TBI, diagnosed on average five years prior to data collection. Accordingly, previous studies indicate that most adolescents, especially after mild TBI, recovered within a few weeks [15,22].

The CFA with ML estimation revealed that the hypothesized four-factor model of the PCSI-SR13 could be replicated. However, a lack of model fit was found for the four-factor structure of the PCSI-P. One explanation for the poor fit of the data from the current parent sample to the hypothesized model may be due to the low parent ratings, which resulted in limited variance in the data (i.e., the response categories were not exhausted). This was also described for parents in the comparison group in the original English study [31].

In the additional CFA with WLSMV estimation using the 7-point Guttman scale, no model fit was obtained for the PCSI-SR13 and the PCSI-P. This was due to the high number of parameters to be estimated in relation to the respective sample size of adolescents and parents of the current study. However, good fit was determined for the four-factor structure of the PCSI-SR13 using aggregated trichotomized response categories.

In addition to the lack of variance in the parent sample of the current study, the different results of the CFA for the German version of the PCSI-SR13 and the PCSI-P may be explained by the time elapsed between the injury and the time of data collection. This suggestion is based on findings from studies indicating that different time periods after TBI have an impact on the factorial structure of P/PCCS assessments [67]. In addition, self-report and parent-report factorial structures may differ depending on the time points at which TBI-related symptom burden is assessed.

Albeit, it should be noted that the sample size of the current study was substantially below the recommended sample of 250 for robust CFA [53]. Therefore, the study CFA results should be interpreted with caution, and further research on factorial validity is indicated.

Overall, internal consistency was found to be good to excellent for both the PCSI- SR13 and the PCSI-P. The correlations between the individual items within their respective scale were, with one exception, at least satisfactory.

For both questionnaire versions, improvements in Cronbach’s α by omitting single items were found to be rather negligible. Although, it is often argued that a higher value in Cronbach’s α is better, it should be kept in mind, that even seemingly unidimensional constructs comprise various aspects [68], as is the case with the subscales of the two questionnaire versions. Therefore, deleting an item could potentially result in a decreased variability of the respective subscale. However, it is important to capture adequately the heterogeneity of outcomes after TBI.

With one exception, all of the Cronbach’s α and McDonald’s ω values for the subscales obtained in this study were similar or even higher than those reported in the original validation study [31]. A considerably lower Cronbach’s α value was observed for the *Cognition* subscale assessed in self-report, although, this value indicated good scale reliability. Based on the current results, it is rather difficult to speculate as to why this difference occurred. Since the value found is comparable to the one reported for the comparison group of the original English study [31], it can only be assumed that one or some of the items do not have the same extent to the subscale in a subacute and chronic phase compared to an acute phase after TBI.

The correlations between the single items with the corresponding subscale and total scale were, except for one item, at least acceptable, overall indicating high internal consistency. For correlation with the total scale the value of the item *Visual Problems* (*double vision, blurring)* was slightly below the assumed cut-off. This result indicates that the item is less relevant for the assessment of PCS in the subacute and chronic phase after TBI. Although visual problems are predominantly reported in the acute phase after TBI [69], recent studies found that in several cases visual problems may persist [70]. Therefore, further investigation on this item for the assessment of PCS in adolescents in a subacute and chronic phase after TBI are required.

Regarding convergent validity, as expected, scales of the PCSI-SR13 and PCSI-P were found to correlate strongly with the RPQ self- and parent-report respectively. However, especially for the parent version, values of the total scale correlations were lower for the current study compared to the values reported for the original English parent version. Overall, higher correlations could have been expected for the current study due to nearly comparable items and wording of the items. However, it can be assumed that due to differences in temporal anchoring of symptom severity ratings of symptoms may lead to different responses. That is, the PCSI versions assess symptom burden for a 2-day-period, whereas the RPQ asks for a rating of the severity of symptom burden compared to the time before TBI. The two-day assessment period of the PCSI-SR13 and PCSI-P post-versions may provide a distinct advantage over the RPQ. This advantage is particularly relevant for evaluating subacute and chronic PCS, as reliable self-report of the pre-TBI status is often difficult to obtain from most individuals, especially if the TBI occurred in early childhood. In such cases, examiners should rely on information reported after injury [31].

In general, item-level agreement between self- and parent-reported P/PCS was only moderate to fair. Nevertheless, subscale level agreement between the adolescents and their parents was good, while the respective ranges of the 95% CI reflected the results found for the item level agreement. The results are also comparable to those reported by Sady et al. [31], who found low to moderate agreement between pediatric participants and parents at the item-level, but moderate to good agreement at the subscale and total scale levels. These findings suggest that in clinical practice, the comparison of agreement between self- and parent-rated symptom burden should not solely be based on the subscale or total scale level. Instead, it is strongly recommended to additionally consider adolescent-parent agreement at the item level. Especially in adolescents, agreement between self- and parent-report has been shown to be considerably low, especially after mild TBI [35], may also vary depending on the type of symptom under consideration [71]. For instance, higher discrepancies between adolescents and their parents were found in the assessment of internal symptom burden (e.g., anxiety) compared to visible symptom burden (e.g., physical impairment) [36]. Furthermore, the smaller sample size may have also contributed to some of the variations noted in this study compared to prior studies in correlating with parental scores.

Comparison of the absolute scale differences revealed that adolescents were more likely to report higher symptom burden on each scale compared to their parents. This result is in line with findings by Hajek et al. [37], who found that the concerned individuals tended to report higher symptom burden than their parents. In addition to this finding, it is also important to consider that disagreement between adolescents and parent ratings increase with the time since injury [72].

Based on our findings, we recommend that especially the *Cognition* subscale should be assessed through self-reports in the subacute and chronic TBI phase.

## Strength and limitations

Although the sample size of participating adolescents and parents of the current study was smaller and more heterogeneous concerning TBI severity compared to the original English study, the results for internal consistency and convergent validity indicate the applicability of the German versions of the PCSI-SR13 and PCSI-P for clinical practice. These validated German versions may help to improve the personalized treatment, especially for adolescents in an outpatient setting, by providing an age-appropriate instrument for P/PCS screening in adolescents in a subacute and chronic TBI phase.

The present study has some limitations to be noted. First, the participation rate of the more than 5000 contacted families only reached 7%, indicating a potential selection bias that primarily depended on the willingness of the parents to participate. Second, most of the participants experienced a mild TBI more than five years prior to study participation.

According to the frequencies reported for P/PCS in adolescents with mild TBI [15,16], it can be assumed that in most cases the symptom burden reported in the current study was due to developmental changes, other complaints rather than TBI, or longer time since injury.

Proportion of females participating in the current study was found to be relatively high, compared to the reports where the proportion of males suffering from pediatric TBI is estimated to be twice that of females [9]. This might have had an impact on the results since it was found that internalizing problems are more likely to be reported by girls compared to boys and agreement with parents ratings tends to be lower [73].

Since the P/PCSI was a secondary outcome in the study project for economic reasons and to reduce patient burden due to the total length of all questionnaires, test-retest assessments were not performed. Therefore, future studies should further investigate the test-retest reliability to evaluate the stability of the underlying PCS construct of both PCSI versions.

Furthermore, the interpretation of the factor analysis results is limited. Due to the right-skewed and non-normal distribution of the responses, a factor analysis could only be conducted after transforming the response scales into three or two categories. Finally, a comparison group of adolescents without a history of TBI would have allowed to evaluate the clinical impact of the post-concussion symptoms better [74].

In conclusion, despite the limitations of the relatively small sample size, the later time of assessment after injury, the lack of test-retest reliability, the study provides initial evidence of the suitability of the German PCSI for assessing PCS in adolescents. Furthermore, the findings offer a valuable contribution to existing literature and hold potential for inclusion in meta-analyses, as synthesizing findings across multiple studies would substantially advance our understanding of TBI in adolescents and provide a more comprehensive assessment of the PCSI’s clinical utility in this population.

## Implications for further research and conclusion

Although the level of agreement between adolescents and their parents was good to moderate at the subscale level, some of the notable discrepancies were partly found at the item level. Therefore, it is recommended that self-report measures of P/PCS are used whenever possible.

To address the limitations of the current study, future research should examine larger sample sizes, more recent TBI cases, and the reliability of both PCSI versions, with a particular focus on the stability of the underlying PCS construct. Furthermore, future research may benefit from the use of longitudinal study designs, as the factorial structure of instruments used to assess P/PCS may vary depending on whether TBI-related symptoms are acute, subacute, or chronic. This would allow this assumption to be tested in more detail and may lead to more specific instruments that account for time-related differences in TBI-related symptom burden. To determine the clinical relevance of the P/PCS, reference values obtained from comparable general pediatric population should be established. This would allow for the identification of developmental effects as well as other proposed physical (e.g., iron-deficiency anemia) or psychological (e.g., depression) symptoms that may correspond to those reported for P/PCS.

The psychometric properties of the German PCSI versions for the assessment of P/PCS in adolescents aged 13–17 years in a subacute and chronic phase of TBI showed high internal consistency and good convergent validity. Therefore, we can recommend their application in research and clinical practice. However, symptoms in adolescents in the subacute and chronic phase after TBI should preferably be assessed by self-report due to the lack of model fit for the parent version and discrepancies in child-parent agreement, especially at the item level.

## Supporting information

S1 Appendix Table A1: Descriptive characteristics for the PCSI-SR13 and PCSI-P Items. (DOCX)

S2 Appendix Table A2: Distribution of item responses on the 7-point Guttman scale. (DOCX)

S3 Appendix Table A3: Comparison of the descriptive characteristics of the current study and those reported by Sady et al. [31]. (DOCX)

S4 Appendix Table B1: German translation of the PSCI-SR13 and PCSI-P. (DOCX)

S1 Questionnaire Inclusivity in global research questionnaire PCSI_SR13. (DOCX)

S1 Checklist PLOS One human subjects research checklist PCSI_SR13. (DOCX)
