# Supplementary material for: German translation and psychometric testing of the Postconcussion Symptom Inventory for adolescents in self-report (PCSI-SR13) and parent-report (PCSI-P)
Source: PLoS One. 2025 Aug 8;20(8):e0307421. doi: 10.1371/journal.pone.0307421 (PMC12333987; doi:10.1371/journal.pone.0307421)
Supplement: S1 Appendix — (DOCX) [file pone.0307421.s001.docx]

# Appendix A

Table A1. Descriptive characteristics for the PCSI-SR13 and PCSI-P Items.

| Sample | Item | Scale | *M* | *SD* | *R* | *SK* | *KU* |
| --- | --- | --- | --- | --- | --- | --- | --- |
| Adolescents  (*N* = 117) | Headache | Physical | 1.18 | 1.46 | 6 | **1.19** | **0.73** |
|  | Nausea | Physical | 0.54 | 1.00 | 5 | 2.37 | 5.89 |
|  | Balance Problems | Physical | 0.73 | 1.19 | 6 | **1.94** | 4.06 |
|  | Dizziness | Physical | 0.74 | 1.21 | 5 | **1.71** | 2.32 |
|  | Visual Problems (double vision, blurring) | Physical | 0.62 | 1.22 | 6 | 2.51 | 6.86 |
|  | Move in a clumsy manner | Physical | 0.74 | 1.01 | 5 | **1.62** | 2.77 |
|  | Sensitivity to light | Physical | 0.50 | 1.07 | 5 | 2.41 | 5.57 |
|  | Sensitivity to noise | Physical | 0.95 | 1.31 | 5 | **1.31** | **0.82** |
|  | Irritability | Emotional | 1.42 | 1.54 | 6 | **1.02** | **0.31** |
|  | Sadness | Emotional | 1.33 | 1.73 | 6 | **1.26** | **0.56** |
|  | Nervousness | Emotional | 1.10 | 1.48 | 6 | **1.44** | **1.53** |
|  | Feeling more emotional | Emotional | 0.94 | 1.46 | 6 | **1.58** | **1.70** |
|  | Feeling mentally `foggy‘ | Cognition | 0.57 | 1.03 | 4 | **1.91** | 2.98 |
|  | Difficulty concentrating | Cognition | 1.50 | 1.49 | 6 | **0.98** | **0.37** |
|  | Difficulty remembering | Cognition | 1.21 | 1.47 | 6 | **1.56** | 2.18 |
|  | Get confused with directions or tasks | Cognition | 1.03 | 1.23 | 6 | **1.43** | 2.19 |
|  | Answers questions more slowly than usual | Cognition | 0.55 | 1.03 | 5 | 2.21 | 4.84 |
|  | Feeling slowed down | Cognition | 0.47 | 1.08 | 6 | 2.95 | 9.35 |
|  | Fatigue | Fatigue | 1.27 | 1.38 | 5 | **0.97** | **0.10** |
|  | Drowsiness | Fatigue | 1.14 | 1.43 | 6 | **1.50** | **1.69** |
|  | Sleep more than usual | Fatigue | 0.76 | 1.26 | 6 | **1.87** | 3.35 |
| Parents  (*N* = 111) | Complains of headaches | Physical | 0.97 | 1.44 | 6 | 1.55 | **1.83** |
|  | Complains of nausea | Physical | 0.28 | 0.82 | 4 | 3.14 | 9.39 |
|  | Has balance problems | Physical | 0.45 | 1.05 | 6 | 3.32 | 13.25 |
|  | Has or complains of dizziness | Physical | 0.44 | 1.16 | 6 | 3.26 | 11.17 |
|  | Has or complains of visual problems (blurry, double vision) | Physical | 0.31 | 0.97 | 6 | 3.91 | 16.69 |
|  | Appears to move in a clumsy  manner | Physical | 0.51 | 1.06 | 6 | 2.69 | 8.57 |
|  | Sensitivity to light | Physical | 0.45 | 1.20 | 6 | 3.18 | 9.91 |
|  | Sensitivity to noise | Physical | 0.66 | 1.30 | 6 | 2.45 | 6.01 |
|  | Acts irritable | Emotional | 1.10 | 1.56 | 6 | **1.54** | 1.95 |
|  | Appears sad | Emotional | 0.68 | 1.45 | 6 | 2.60 | 6.22 |
|  | Acts nervous | Emotional | 0.55 | 1.20 | 6 | 2.78 | 7.88 |
|  | Acts more emotional | Emotional | 1.12 | 1.67 | 6 | **1.68** | 2.15 |
|  | Acts or appears mentally `foggy` | Cognition | 0.23 | 0.81 | 5 | 4.03 | 17.03 |
|  | Has difficulty concentrating | Cognition | 1.23 | 1.63 | 6 | **1.18** | **0.28** |
|  | Has difficulty remembering | Cognition | 0.69 | 1.39 | 6 | 2.04 | 3.20 |
|  | Becomes confused with directions or tasks | Cognition | 0.58 | 1.13 | 5 | 2.11 | 3.78 |
|  | Answers questions more slowly than usual | Cognition | 0.48 | 1.14 | 6 | 2.70 | 7.57 |
|  | Appears more tired or fatigued | Fatigue | 0.96 | 1.51 | 6 | 1.64 | **1.85** |
|  | Appears drowsy | Fatigue | 0.60 | 1.29 | 6 | 2.48 | 5.69 |
|  | Sleeping more than usual | Fatigue | 0.60 | 1.30 | 6 | 2.38 | 5.14 |

*n* = Sample size, *M* = Mean, *SD* = Standard deviation, *SK* = Skewness, *KU* = Kurtosis. Values in bold indicate acceptable *SK* and *KU* (-2 to +2).
