# Supplementary material for: German translation and psychometric testing of the Postconcussion Symptom Inventory for adolescents in self-report (PCSI-SR13) and parent-report (PCSI-P)
Source: PLoS One. 2025 Aug 8;20(8):e0307421. doi: 10.1371/journal.pone.0307421 (PMC12333987; doi:10.1371/journal.pone.0307421)
Supplement: S2 Appendix — (DOCX) [file pone.0307421.s002.docx]

# Appendix A

Table A2. Distribution of item responses on the 7-point Guttman scale.

| **Item** | **Response category** | **Self-report** | **Parent-report** |
| --- | --- | --- | --- |
|  |  | **PCSI-SR13** | **PCSI-P** |
|  |  | *N* = 117 (%) | *N* = 111 (%) |
|  |  | *N* (%) | *N* (%) |
| Headache | 0 | 56 (47.9%) | 64 (57.7%) |
|  | 1 | 21 (17.9%) | 18 (16.2%) |
|  | 2 | 18 (15.4%) | 10 (9.0%) |
|  | 3 | 13 (11.1%) | 11 (9.9%) |
|  | 4 | 4 (3.4%) | 5 (4.5%) |
|  | 5 | 4 (3.4%) | 1 (0.9%) |
|  | 6 | 1 (0.9%) | 2 (1.8%) |
| Nausea | 0 | 79 (67.5%) | 97 (87.4%) |
|  | 1 | 25 (21.4%) | 4 (3.6%) |
|  | 2 | 6 (5.1%) | 5 (4.5%) |
|  | 3 | 3 (2.6%) | 3 (2.7%) |
|  | 4 | 3 (2.6%) | 2 (1.8%) |
|  | 5 | 1 (0.9%) | 0 (0.0%) |
|  | 6 | 0 (0.0%) | 0 (0.0%) |
| Balance problems | 0 | 74 (63.2%) | 85 (76.6%) |
|  | 1 | 18 (15.4%) | 13 (11.7%) |
|  | 2 | 15 (12.8%) | 8 (7.2%) |
|  | 3 | 6 (5.1%) | 3 (2.7%) |
|  | 4 | 2 (1.7%) | 0 (0.0%) |
|  | 5 | 1 (0.9%) | 0 (0.0%) |
|  | 6 | 1 (0.9%) | 2 (1.8%) |
| Dizziness | 0 | 74 (63.2%) | 90 (81.1%) |
|  | 1 | 19 (16.2%) | 9 (8.1%) |
|  | 2 | 11 (9.4%) | 5 (4.5%) |
|  | 3 | 8 (6.8%) | 3 (2.7%) |
|  | 4 | 3 (2.6%) | 1 (0.9%) |
|  | 5 | 2 (1.7%) | 1 (0.9%) |
|  | 6 | 0 (0.0%) | 2 (1.8%) |
| Visual problems  (double vision, blurring) | 0 | 82 (70.1%) | 97 (87.4%) |
|  | 1 | 15 (12.8%) | 4 (3.6%) |
|  | 2 | 12 (10.3%) | 6 (5.4%) |
|  | 3 | 3 (2.6%) | 1 (0.9%) |
|  | 4 | 2 (1.7%) | 1 (0.9%) |
|  | 5 | 1 (0.9%) | 1 (0.9%) |
|  | 6 | 2 (1.7%) | 1 (0.9%) |
|  |  |  |  |
|  |  |  |  |
|  |  |  |  |
| Move in a clumsy manner | 0 | 63 (53.8%) | 83 (74.8%) |
|  | 1 | 34 (29.1%) | 11 (9.9%) |
|  | 2 | 11 (9.4%) | 11 (9.9%) |
|  | 3 | 7 (6.0%) | 4 (3.6%) |
|  | 4 | 1 (0.9%) | 0 (0.0%) |
|  | 5 | 1 (0.9%) | 1 (0.9%) |
|  | 6 | 0 (0.0%) | 1 (0.9%) |
| Sensitivity to light | 0 | 90 (76.9%) | 90 (81.1%) |
|  | 1 | 10 (8.5%) | 10 (9.0%) |
|  | 2 | 8 (6.8%) | 4 (3.6%) |
|  | 3 | 6 (5.1%) | 2 (1.8%) |
|  | 4 | 1 (0.9%) | 0 (0.0%) |
|  | 5 | 2 (1.7%) | 4 (3.6%) |
|  | 6 | 0 (0.0%) | 1 (1%) |
| Sensitivity to noise | 0 | 65 (55.6%) | 77 (69.4%) |
|  | 1 | 19 (16.2%) | 17 (15.3%) |
|  | 2 | 17 (14.5%) | 6 (5.4%) |
|  | 3 | 8 (6.8%) | 6 (5.4%) |
|  | 4 | 6 (5.1%) | 1 (0.9%) |
|  | 5 | 2 (1.7%) | 2 (1.8%) |
|  | 6 | 0 (0.0%) | 2 (1.8%) |
| Irritability | 0 | 44 (37.6%) | 61 (55.0%) |
|  | 1 | 29 (24.8%) | 15 (13.5%) |
|  | 2 | 15 (12.8%) | 15 (13.5%) |
|  | 3 | 17 (14.5%) | 13 (11.7%) |
|  | 4 | 6 (5.1%) | 1 (0.9%) |
|  | 5 | 4 (3.4%) | 2 (1.8%) |
|  | 6 | 2 (1.7%) | 4 (3.6%) |
| Sadness | 0 | 57 (48.7%) | 79 (71.2%) |
|  | 1 | 20 (17.1%) | 17 (15.3%) |
|  | 2 | 16 (13.7%) | 5 (4.5%) |
|  | 3 | 7 (6.0%) | 2 (1.8%) |
|  | 4 | 7 (6.0%) | 2 (1.8%) |
|  | 5 | 6 (5.1%) | 2 (1.8%) |
|  | 6 | 4 (3.4%) | 4 (3.6%) |
| Nervousness | 0 | 59 (50.4%) | 81 (73.0%) |
|  | 1 | 23 (19.7%) | 17 (15.3%) |
|  | 2 | 16 (13.7%) | 5 (4.5%) |
|  | 3 | 9 (7.7%) | 3 (2.7%) |
|  | 4 | 5 (4.3%) | 1 (0.9%) |
|  | 5 | 3 (2.6%) | 3 (2.7%) |
|  | 6 | 2 (1.7%) | 1 (0.9%) |
|  |  |  |  |
|  |  |  |  |
|  |  |  |  |
| Feeling more emotional | 0 | 71 (60.7%) | 61 (55.0%) |
|  | 1 | 15 (12.8%) | 18 (16.2%) |
|  | 2 | 14 (12.0%) | 14 (12.6%) |
|  | 3 | 7 (6.0%) | 8 (7.2%) |
|  | 4 | 5 (4.3%) | 2 (1.8%) |
|  | 5 | 4 (3.4%) | 2 (1.8%) |
|  | 6 | 1 (0.9%) | 6 (5.4%) |
| Feeling mentally `foggy’ | 0 | 81 (69.2%) | 101 (91.0%) |
|  | 1 | 17 (14.5%) | 2 (1.8%) |
|  | 2 | 11 (9.4%) | 4 (3.6%) |
|  | 3 | 4 (3.4%) | 2 (1.8%) |
|  | 4 | 4 (3.4%) | 1 (0.9%) |
|  | 5 | 0 (0.0%) | 1 (0.9%) |
|  | 6 | 0 (0.0%) | 0 (0.0%) |
| Difficulty concentrating | 0 | 36 (30.8%) | 57 (51.4%) |
|  | 1 | 34 (29.1%) | 18 (16.2%) |
|  | 2 | 20 (17.1%) | 11 (9.9%) |
|  | 3 | 13 (11.1%) | 12 (10.8%) |
|  | 4 | 9 (7.7%) | 5 (4.5%) |
|  | 5 | 3 (2.6%) | 7 (6.3%) |
|  | 6 | 2 (1.7%) | 1 (0.9%) |
| Difficulty remembering | 0 | 47 (40.2%) | 82 (73.9%) |
|  | 1 | 35 (29.9%) | 9 (8.1%) |
|  | 2 | 19 (16.2%) | 6 (5.4%) |
|  | 3 | 5 (4.3%) | 4 (3.6%) |
|  | 4 | 5 (4.3%) | 7 (6.3%) |
|  | 5 | 3 (2.6%) | 2 (1.8%) |
|  | 6 | 3 (2.6%) | 1 (0.9%) |
| Get confused with directions or tasks | 0 | 51 (43.6%) | 80 (72.1%) |
|  | 1 | 35 (29.9%) | 14 (12.6%) |
|  | 2 | 16 (13.7%) | 7 (6.3%) |
|  | 3 | 10 (8.5%) | 5 (4.5%) |
|  | 4 | 3 (2.6%) | 4 (3.6%) |
|  | 5 | 1 (0.9%) | 1 (0.9%) |
|  | 6 | 1 (0.9%) | 0 (0.0%) |
| Answer questions more slowly than usual | 0 | 82 (70.1%) | 90 (81.1%) |
|  | 1 | 18 (15.4%) | 3 (2.7%) |
|  | 2 | 10 (8.5%) | 11 (9.9%) |
|  | 3 | 3 (2.6%) | 3 (2.7%) |
|  | 4 | 3 (2.6%) | 2 (1.8%) |
|  | 5 | 1 (0.9%) | 1 (0.9%) |
|  | 6 | 0 (0.0%) | 1 (0.9%) |
|  |  |  |  |
|  |  |  |  |
|  |  |  |  |
| Feeling slowed down^a^ | 0 | 89 (76.1%) | − − − |
|  | 1 | 16 (13.7%) | − − − |
|  | 2 | 4 (3.4%) | − − − |
|  | 3 | 4 (3.4%) | − − − |
|  | 4 | 2 (1.7%) | − − − |
|  | 5 | 1 (0.9%) | − − − |
|  | 6 | 1 (0.9%) | − − − |
| Fatigue | 0 | 45 (38.5%) | 67 (60.4%) |
|  | 1 | 33 (28.2%) | 14 (12.6%) |
|  | 2 | 13 (11.1%) | 15 (13.5%) |
|  | 3 | 18 (15.4%) | 5 (4.5%) |
|  | 4 | 4 (3.4%) | 3 (2.7%) |
|  | 5 | 4 (3.4%) | 6 (5.4%) |
|  | 6 | 0 (0.0%) | 1 (0.9%) |
| Drowsiness | 0 | 50 (42.7%) | 83 (74.8%) |
|  | 1 | 36 (30.8%) | 12 (10.8%) |
|  | 2 | 14 (12.0%) | 6 (5.4%) |
|  | 3 | 6 (5.1%) | 3 (2.7%) |
|  | 4 | 5 (4.3%) | 3 (2.7%) |
|  | 5 | 5 (4.3%) | 3 (2.7%) |
|  | 6 | 1 (0.9%) | 1 (0.9%) |
| Sleep more than usual | 0 | 75 (64.1%) | 84 (75.7%) |
|  | 1 | 15 (12.8%) | 10 (9.0%) |
|  | 2 | 17 (14.5%) | 5 (4.5%) |
|  | 3 | 3 (2.6%) | 6 (5.4%) |
|  | 4 | 5 (4.3%) | 2 (1.8%) |
|  | 5 | 1 (0.9%) | 3 (2.7%) |
|  | 6 | 1 (0.9%) | 1 (0.9%) |

0 = not a problem, 3 = moderate problem, 6 = severe Problem. ^a^ No equivalent item in the parent version.
