# Supplementary material for: German translation and psychometric testing of the Postconcussion Symptom Inventory for adolescents in self-report (PCSI-SR13) and parent-report (PCSI-P)
Source: PLoS One. 2025 Aug 8;20(8):e0307421. doi: 10.1371/journal.pone.0307421 (PMC12333987; doi:10.1371/journal.pone.0307421)
Supplement: S3 Appendix — (DOCX) [file pone.0307421.s003.docx]

# Appendix A

Table A3. Comparison of the descriptive characteristics of the current study and those reported by Sady et al. [32].

| Subscale | Outcome |  | Self-report  PCSI-SR13 | | Parent-report  PCSI-P | |
| --- | --- | --- | --- | --- | --- | --- |
|  | | Rating | Present study  (*N* = 117) | Study Sady et al., 2014  *(N* = 223) | Present study  (*N* = 111) | Study Sady et al., 2014  (*N* = 521) |
| Physical | Headache | *M* (*SD*) | **1.18** (1.46) | **2.38** (1.93) | **0.97** (1.44) | **2.70** (2.07) |
|  |  | 0 (%) | 48 | 27 | 58 | 24 |
|  |  | 1-3 (%) | 45 | 39 | 35 | 39 |
|  |  | 4-6 (%) | 8 | 34 | 7 | 37 |
|  | Nausea | *M* (*SD*) | 0.54 (1.00) | 0.49 (1.10) | 0.28 (0.82) | 0.90 (1.57) |
|  |  | 0 (%) | 68 | 79 | 87 | 68 |
|  |  | 1-3 (%) | 29 | 18 | 10 | 22 |
|  |  | 4-6 (%) | 3 | 3 | 2 | 10 |
|  | Balance  Problems | *M* (*SD*) | 0.73 (1.19) | 0.61 (1.26) | 0.45 (1.05) | 0.91 (1.49) |
|  |  | 0 (%) | 63 | 75 | 77 | 64 |
|  |  | 1-3 (%) | 33 | 18 | 22 | 27 |
|  |  | 4-6 (%) | 3 | 6 | 2 | 9 |
|  | Dizziness | *M* (*SD*) | 0.74 (1.21) | 1.00 (1.54) | 0.44 (1.16) | 1.21 (1.72) |
|  |  | 0 (%) | 63 | 64 | 81 | 57 |
|  |  | 1-3 (%) | 33 | 26 | 15 | 31 |
|  |  | 4-6 (%) | 4 | 11 | 4 | 12 |
|  | Visual  problems  (double vision,  blurring) | *M* (*SD*) | 0.62 (1.22) | 0.55 (1.17) | 0.31 (0.97) | 0.74 (1.42) |
|  |  | 0 (%) | 70 | 77 | 87 | 72 |
|  |  | 1-3 (%) | 26 | 18 | 10 | 21 |
|  |  | 4-6 (%) | 4 | 5 | 3 | 7 |
|  | Move in a clumsy manner | *M* (*SD*) | 0.74 (1.01) | 1.12 (1.60) | 0.51 (1.06) | 0.59 (1.24) |
|  |  | 0 (%) | 54 | 55 | 75 | 74 |
|  |  | 1-3 (%) | 44 | 34 | 23 | 21 |
|  |  | 4-6 (%) | 2 | 12 | 2 | 5 |
|  | Sensitivity to light | *M* (*SD*) | 0.50 (1.07) | 1.25 (1.70) | 0.45 (1.20) | 1.27 (1.72) |
|  |  | 0 (%) | 77 | 59 | 81 | 57 |
|  |  | 1-3 (%) | 21 | 26 | 14 | 28 |
|  |  | 4-6 (%) | 3 | 16 | 5 | 15 |
|  |  |  |  |  |  |  |
| Physical | Sensitivity to noise | *M* (*SD*) | 0.95 (1.31) | 0.94 (1.64) | 0.66 (1.30) | 1.22 (1.78) |
|  |  | 0 (%) | 56 | 69 | 69 | 60 |
|  |  | 1-3 (%) | 38 | 20 | 26 | 24 |
|  |  | 4-6 (%) | 7 | 11 | 5 | 16 |
| Emotional | Irritability | *M* (*SD*) | 1.42 (1.54) | 0.94 (1.59) | 1.10 (1.56) | 1.45 (1.78) |
|  |  | 0 (%) | 38 | 67 | 55 | 47 |
|  |  | 1-3 (%) | 52 | 23 | 39 | 38 |
|  |  | 4-6 (%) | 10 | 10 | 6 | 15 |
|  | Sadness | *M* (*SD*) | **1.33** (1.73) | **0.50** (1.27) | 0.68 (1.45) | 0.92 (1.45) |
|  |  | 0 (%) | 49 | 83 | 71 | 63 |
|  |  | 1-3 (%) | 37 | 12 | 22 | 30 |
|  |  | 4-6 (%) | 15 | 6 | 7 | 8 |
|  | Nervousness | *M* (*SD*) | **1.10** (1.48) | **0.48** (1.15) | 0.55 (1.20) | 0.58 (1.25) |
|  |  | 0 (%) | 50 | 79 | 73 | 76 |
|  |  | 1-3 (%) | 41 | 18 | 23 | 18 |
|  |  | 4-6 (%) | 9 | 3 | 5 | 5 |
|  | Feeling more emotional | *M* (*SD*) | 0.94 (1.46) | 0.59 (1.38) | 1.12 (1.67) | 1.19 (1.72) |
|  |  | 0 (%) | 61 | 80 | 55 | 57 |
|  |  | 1-3 (%) | 31 | 14 | 36 | 29 |
|  |  | 4-6 (%) | 9 | 6 | 9 | 14 |
| Cognition | Feeling mentally ‘foggy’ | *M* (*SD*) | 0.57 (1.03) | 1.41 (1.93) | **0.23** (0.81) | **1.25** (1.69) |
|  |  | 0 (%) | 69 | 58 | 91 | 53 |
|  |  | 1-3 (%) | 27 | 19 | 7 | 35 |
|  |  | 4-6 (%) | 3 | 23 | 2 | 12 |
|  | Difficulty concentrating | *M* (*SD*) | 1.50 (1.49) | 1.98 (2.05) | 1.23 (1.63) | 1.74 (1.90) |
|  |  | 0 (%) | 31 | 42 | 51 | 42 |
|  |  | 1-3 (%) | 58 | 29 | 37 | 39 |
|  |  | 4-6 (%) | 12 | 29 | 12 | 19 |
|  | Difficulty remembering | *M* (*SD*) | 1.21 (1.47) | 1.47 (1.86) | 0.69 (1.39) | 1.32 (1.74) |
|  |  | 0 (%) | 40 | 51 | 74 | 50 |
|  |  | 1-3 (%) | 50 | 32 | 17 | 37 |
|  |  | 4-6 (%) | 9 | 17 | 9 | 13 |
|  | Get confused with directions and tasks | *M* (*SD*) | 1.03 (1.23) | 1.59 (1.68) | 0.58 (1.13) | 0.93 (1.54) |
|  |  | 0 (%) | 44 | 40 | 72 | 64 |
|  |  | 1-3 (%) | 52 | 43 | 23 | 27 |
|  |  | 4-6 (%) | 4 | 17 | 5 | 9 |
|  |  |  |  |  |  |  |
| Cognition | Answer questions more slowly than usual | *M* (*SD*) | **0.55** (1.03) | **1.76** (1.83) | 0.48 (1.14) | 0.98 (1.55) |
|  |  | 0 (%) | 70 | 37 | 81 | 60 |
|  |  | 1-3 (%) | 27 | 42 | 15 | 31 |
|  |  | 4-6 (%) | 3 | 21 | 4 | 9 |
|  | Feeling slowed down | *M* (*SD*) | 0.47 (1.08) | 1.40 (1.86) | − − − | − − − |
|  |  | 0 (%) | 76 | 57 | − − − | − − − |
|  |  | 1-3 (%) | 21 | 26 | − − − | − − − |
|  |  | 4-6 (%) | 3 | 17 | − − − | − − − |
| Fatigue | Fatigue | *M* (*SD*) | 1.27 (1.38) | 1.94 (1.96) | 0.96 (1.51) | 1.93 (1.94) |
|  |  | 0 (%) | 39 | 39 | 60 | 36 |
|  |  | 1-3 (%) | 55 | 32 | 31 | 41 |
|  |  | 4-6 (%) | 7 | 28 | 9 | 23 |
|  | Drowsiness | *M* (*SD*) | 1.14 (1.43) | 1.70 (1.88) | 0.60 (1.29) | 1.30 (1.70) |
|  |  | 0 (%) | 43 | 45 | 75 | 53 |
|  |  | 1-3 (%) | 48 | 36 | 19 | 34 |
|  |  | 4-6 (%) | 9 | 19 | 6 | 13 |
|  | Sleep more than usual | *M* (*SD*) | 0.76 (1.26) | 1.08 (1.81) | 0.60 (1.30) | 1.51 (1.91) |
|  |  | 0 (%) | 64 | 68 | 76 | 51 |
|  |  | 1-3 (%) | 30 | 17 | 19 | 31 |
|  |  | 4-6 (%) | 6 | 15 | 5 | 18 |

*n* = number of observations in the present study, *N* = number of observations in the study by Sady et al.,2014, *M* = mean, *SD* = standard deviation, % = percentage of responses per grouped category. Means that differ more than half a *SD* as reported in the English validation study are highlighted in bold. Due to rounding, not all percentages sum up to 100%.
