# Supplementary material for: German translation and psychometric testing of the Postconcussion Symptom Inventory for adolescents in self-report (PCSI-SR13) and parent-report (PCSI-P)
Source: PLoS One. 2025 Aug 8;20(8):e0307421. doi: 10.1371/journal.pone.0307421 (PMC12333987; doi:10.1371/journal.pone.0307421)
Supplement: S4 Appendix — (DOCX) [file pone.0307421.s004.docx]

**Appendix B**

**Table B 1. German translation of the PSCI-SR13 and PCSI-P.**

|  | PCSI-SR 13 | PCSI-P |
| --- | --- | --- |
| Introduction | Wir möchten wissen, ob Du nach Deiner Hirnverletzung irgendeine dieser Beschwerden hast. Bitte beantworte alle Fragen so gut Du kannst. Lasse keine Frage aus. Markiere diejenige Zahl, die uns zeigt, wie problematisch die Beschwerden für Dich aktuell (gestern und heute) sind. | Wir möchten wissen, ob Ihr Kind nach seiner Hirnverletzung irgendeine der folgenden Beschwerden hat. Bitte beantworten Sie alle Fragen so gut Sie können. Lassen Sie keine Frage aus. Markieren Sie die entsprechende Zahl, um uns mitzuteilen, wie problematisch die Beschwerden für Ihr Kind aktuell (gestern und heute) sind. |
| Anchor responses | 0 = Kein Problem | 0 = Kein Problem |
|  | 3 = Mittleres Problem | 3 = Mittleres Problem |
|  | 6 = Schwerwiegendes Problem | 6 = Schwerwiegendes Problem |
| Items | Kopfschmerzen | Klagt über Kopfschmerzen |
|  | Übelkeit | Klagt über Übelkeit |
|  | Probleme mit dem Gleichgewicht | Hat Probleme mit dem Gleichgewicht |
|  | Schwindelgefühle | Zeigt oder klagt über Schwindel |
|  | Probleme mit dem Sehen (verschwommen sehen, doppelt sehen/ Doppelbilder) | Hat oder klagt über Probleme mit dem Sehen (Verschwommen sehen, doppelt sehen/ Doppelbilder) |
|  | Ungeschickte Bewegungen | Wirkt in Bewegungen ungeschickt |
|  | Lichtempfindlichkeit | Lichtempfindlichkeit |
|  | Geräusch-/ Lärmempfindlichkeit | Geräusch-/ Lärmempfindlichkeit |
|  | Reizbarkeit | Verhielt sich reizbar |
|  | Traurigkeit | Wirkt traurig |
|  | Nervosität | Wirkt nervös |
|  | Gefühl, dünnhäutiger (emotionaler) zu sein | Wirkt dünnhäutiger (emotionaler) |
|  | Sich "benebelt" fühlen | Verhält sich oder erscheint "benebelt" |
|  | Schwierigkeiten, sich zu konzentrieren | Hat Schwierigkeiten, sich zu konzentrieren |
|  | Schwierigkeiten, sich zu erinnern | Hat Schwierigkeiten, sich zu erinnern |
|  | Durch Anweisungen oder Aufgaben verwirrt werden | Wird durch Anweisungen oder Aufgaben verwirrt |
|  | Fragen langsamer beantworten als gewöhnlich | Beantwortet Fragen langsamer als gewöhnlich |
|  | Gefühl, verlangsamt zu sein | − − − |
|  | Erschöpfung | Wirkt müder oder erschöpfter |
|  | Schläfrigkeit | Wirkt schläfrig |
|  | Mehr als gewöhnlich schlafen | Schläft mehr als gewöhnlich |
